# Harnessing Reddit to Understand the Written-Communication Challenges Experienced by Individuals With Mental Health Disorders: Analysis of Texts From Mental Health Communities

**DOI:** 10.2196/jmir.8219

**Published:** 2018-04-10

**Authors:** Albert Park, Mike Conway

**Affiliations:** ^1^ Department of Biomedical Informatics School of Medicine University of Utah Salt Lake City, UT United States

**Keywords:** mental health, depression, depressive disorder, major, depressive disorder, bipolar disorder, bipolar, bipolar and related disorders, schizophrenia, schizotypal personality disorder, schizophrenia spectrum and other psychotic disorders, consumer health information, informatics, information science, social support, psychosocial support system, community networks, self-help groups, communications media

## Abstract

**Background:**

Mental disorders such as depression, bipolar disorder, and schizophrenia are common, incapacitating, and have the potential to be fatal. Despite the prevalence and gravity of mental disorders, our knowledge concerning everyday challenges associated with them is relatively limited. One of the most studied deficits related to everyday challenges is language impairment, yet we do not know how mental disorders can impact common forms of written communication, for example, social media.

**Objective:**

The aims of this study were to investigate written communication challenges manifest in online mental health communities focusing on depression, bipolar disorder, and schizophrenia, as well as the impact of participating in these online mental health communities on written communication. As the control, we selected three online health communities focusing on positive emotion, exercising, and weight management.

**Methods:**

We examined lexical diversity and readability, both important features for measuring the quality of writing. We used four well-established readability metrics that consider word frequencies and syntactic complexity to measure writers’ written communication ability. We then measured the lexical diversity by calculating the percentage of unique words in posts. To compare lexical diversity and readability among communities, we first applied pairwise independent sample *t* tests, followed by *P* value adjustments using the prespecified Hommel procedure to adjust for multiple comparison. To measure the changes, we applied linear least squares regression to the readability and lexical diversity scores against the interaction sequence for each member, followed by pairwise independent sample *t* tests and *P* value adjustments. Given the large sample of members, we also report effect sizes and 95% CIs for the pairwise comparisons.

**Results:**

On average, members of depression, bipolar disorder, and schizophrenia communities showed indications of difficulty expressing their ideas compared with three other online health communities. Our results also suggest that participating in these platforms has the potential to improve members’ written communication. For example, members of all three mental health communities showed statistically significant improvement in both lexical diversity and readability compared with members of the OHC focusing on positive emotion.

**Conclusions:**

We provide new insights into the written communication challenges faced by individuals suffering from depression, bipolar disorder, and schizophrenia. A comparison with three other online health communities suggests that written communication in mental health communities is significantly more difficult to read, while also consisting of a significantly less diverse lexicon. We contribute practical suggestions for utilizing our findings in Web-based communication settings to enhance members’ communicative experience. We consider these findings to be an important step toward understanding and addressing everyday written communication challenges among individuals suffering from mental disorders.

## Introduction

Mental disorders are common, incapacitating [1], and account for many years of lost productivity [2]. In addition, serious mental disorders [3] such as depression [4], bipolar disorder [5], and schizophrenia [6] have the potential to be fatal because of the increased risk of suicide. Despite the prevalence and gravity of mental disorders, our knowledge concerning everyday challenges associated with these conditions is relatively limited, especially when compared with many physical conditions.

One of the most studied deficits related to everyday challenges for individuals suffering from depression, bipolar disorder, and schizophrenia is language impairment [7-12]. Researchers of these mental disorders have long suspected language impairment because of deficits in frontal lobe functioning [10,13], which controls both emotion regulation and language processing. Language impairment is typically measured through one’s performance in semantic processing tasks (ie, determining semantic relationships between a word, phrase, or category [14-16] or differentiating real words from pseudowords [17,18] based on an individual’s semantic network [19]) and verbal fluency tasks (ie, production of words from phonemic or semantic categories [20-22]). Despite the importance of language in everyday life, these studies do not illustrate daily challenges associated with language impairment. Moreover, generalizability remains uncertain because of small sample size [7,8], with inconsistent results regarding language impairment or frontal lobe activities [10,23,24].

Despite its potential for devastating disability, it is unclear how language impairment manifests in common forms of written communication, for example, social media communication. With increasing use of technology comes increasing opportunity to write. For instance, in 2015, 84% of American adults used the internet [25], and one of the most frequent uses of the internet is written communication [26], including communication on social media. Nearly two-thirds of American adults use social media, roughly a tenfold increase from a decade ago [27]. A few social media platforms and online mental health communities within Reddit, for example, have become a popular venue for individuals suffering from mental disorders [28]. Reddit supports throwaway and unidentifiable accounts, which can protect users from social discrimination surrounding mental disorders [29-31] and allow honest discussions that may not be appropriate on other social media sites such as Facebook [32]. Reddit also provides contextual information that is relatively limited in other popular social media platforms (eg, Twitter), because of length limitations.

It is also known that effortful tasks (ie, requiring attention) such as expressing thoughts via writing are more difficult than automatic tasks (ie, not requiring attention) for individuals suffering from depression and bipolar disorder, whereas both types of tasks are equally difficult for schizophrenia patients [33,34]. From previous studies on mental disorders and Reddit [30,35-37], we can infer that individuals suffering from mental disorders also frequently engage in written communication, yet the written communication challenges faced by individuals in online mental health communities remain unknown.

Examining important features of writing provides an opportunity to assess members’ written communication skills and any associated linguistic challenges. For example, a study on writing quality used linguistic features such as lexical diversity, syntactic complexity, and word frequency to predict the quality of writing [38]. In different studies, ease of reading (ie, simple and clear writing) [39] and text cohesion with respect to text flow [40] were suggested as some of the most determinant features of writing quality.

We can examine these features to assess online mental health community members’ written communication challenges. More specifically, less lexical diversity and poor readability in posts can be a sign of language impairment. Research on language impairment has linked significantly less lexical diversity with specific language impairments [41]. Similarly, poor sentence structure and difficulties with organization and articulating ideas, which can be described as insufficient readability [42], were also associated with language impairment [43].

Readability metrics have been long-studied or used in the field of communication [39,44], education [45], and informatics [46-58], including social media writing [58]. Readability metrics provide quantitative estimates of the ease with which readers can comprehend a written text. Typically, they are given as an estimated US grade level by measuring the linguistic characteristics of a given text [59]. Moreover, readability metrics, although rudimentary, consider two of the three aforementioned features associated with writing quality: word frequencies [39,57,59,60] and syntactic complexity [38,39,45,57,59-61]. From the perspectives of the writers and their writing quality, readability metrics can measure the writers’ ability to present ideas simply in a straightforward manner. According to one of the developers of the readability metrics, higher readability scores can indicate needless complexity [44] or writing challenges, such as organization and articulating ideas. Language impairment can hinder writers’ ability to simply articulate ideas with ease, while using a less diverse lexicon. Moreover, one benefit of using these readability metrics is that they are computationally simple and relatively straightforward to apply. Thus, we use readability along with lexical diversity (ie, the third writing quality feature) of posts as a proxy for written communication challenges among individuals suffering from depression, bipolar disorder, and schizophrenia.

Though mental health and language impairment have been studied extensively [7-12], less is known about written communication challenges manifested in social media, as well as the effects of long-term participation in online mental health communities on written communication challenges among individuals suffering from depression, bipolar disorder, and schizophrenia disorder. Understanding written communication challenges among these individuals has implications for treating mental disorders, managing online mental health communities, and conducting future research. Despite the importance in clinical, practical, and public policy implications for mental health, to our knowledge, the investigation of written communication challenges utilizing communication in online mental health communities has not been the focus of previous research on mental health.

We aim to fill this gap in the literature with this study and address two research questions (RQ):

RQ1: To what extent do written communication challenges manifest in online mental health communities focusing on depression, bipolar disorder, and schizophrenia? As the control, we selected three online health communities (OHCs): one with less emotional challenges and two with less medical or technical terminology.

RQ2: How would acts of participation (ie, posting to interact with other members) in online mental health communities impact members’ written communication?

## Methods

### Community Platform

The data for this study consist of submissions and their associated comments from Reddit’s several topically focused subcommunities called subreddits. Submissions are posts that start a conversation, and comments are posts that reply to submissions or other comments. Reddit is a highly popular social media platform with more than 82.5 billion page views, 73 million submissions, and 725 million associated comments from 88,700 active subreddits in 2015 [62]. In addition to Reddit’s popularity, Reddit has features suitable for protecting mental health community members’ identity (eg, throwaway and unidentifiable accounts). Thus, we examined submissions and comments (*posts* from here on out to maintain clarity) from Reddit to investigate written communication challenges among individuals suffering from potentially stigmatized conditions.

### Subreddit Selection

r/depression, r/bipolar, and r/schizophrenia, to our knowledge, are the largest and most active subreddits for their respective mental disorders [63-65]. In May 2017, r/depression has been active for 8 years with 178,921 subscribers [63], r/bipolar has 24,724 subscribers and was formed 8 years ago [64], and r/schizophrenia has 7036 subscribers and has been active for 7 years [65]. Thus, we selected r/depression, r/bipolar, and r/schizophrenia as the main communities of interest for investigating the written communication challenges faced by individuals in online mental health communities.

To understand the significance of written communication among r/depression, r/bipolar, and r/schizophrenia members, we selected r/happy [66], r/loseit [67], and r/bodybuilding [68] for the controls. We first selected r/happy, a subreddit that was created to share positive thoughts and happy stories. The subreddit has been active for 9 years with 116,441 subscribers as of May 2017 [66]. Members of most OHCs experience emotional challenges [69-71] from the distress of living with—or being diagnosed with—a serious condition. However, we looked for an OHC that is not directly related to mental disorders, especially depression, to help ensure that this control group’s written communication challenges are not related to mental distress even as a secondary symptom. Thus, we selected the largest and most active, positive, emotion-focused subreddit in Reddit.

We selected a second OHC, r/loseit, to bolster the quality of our findings. r/loseit is a subreddit focusing on weight management and has been a community for 6 years with 425,934 subscribers [67]. We purposely selected a community without a substantial amount of medical or technical terminology because a high level of difficult medical or technical terminology can skew the readability of posts. Although it may be impossible to select OHCs without any medical or technical terminology, one study of r/loseit characterized the most-discussed topics of the community as ordinary health information and management strategies, which can be described without complex medical or technical terminology (eg, food, clothing, physical appearance, workouts, and calorie counting) [72]. Moreover, unlike r/happy, r/loseit contains a substantial amount of emotional support [73], which can indicate that the members are facing emotional challenges similar to many OHCs. Thus, we selected r/loseit, the largest weight management community in Reddit, as a second control group.

We selected a third OHC, r/bodybuilding, in which members are dedicated to passion-centric activities, exercising, and muscular development. The bodybuilding community has 259,743 subscribers and has been active for 9 years [68]. A previous study suggested that members of an online bodybuilding community exchange a considerable level of emotional support (eg, motivational support and competition preparation support) and informational support (eg, training regimes and diets) [74]. The general discussion topics among bodybuilding community members could be relatively similar to the discussion topics among members of r/loseit; however, the two communities could consist of vastly different individuals with respect to health-related goals and habits. Thus, we include r/bodybuilding, the largest and most active muscular development community in Reddit, as the last control group.

### Data

First, we used a dataset [75] (publicly available posts from October 2007 to May 2015) that was collected and archived by a Reddit member and has been used in several previous studies [36,76,77]. Second, we extracted posts made in r/depression, r/bipolar, r/schizophrenia, r/happy, r/loseit, and r/bodybuilding. We excluded posts that were marked as *[deleted* *]* in our analyses. Third, we removed posts with less than five words to help ensure the posts have expressive content and thoughts. Many posts in online communities are short—for example, one-word answering posts (eg, “yes” and “sure”) that can be viewed as automatic tasks rather than effortful tasks. These posts can skew the results; thus, we removed posts with less than five words. Fourth, to restrict our investigation to regular members (ie, exclude throwaway accounts or infrequent members) of the communities, we confined our analysis to members (ie, unique member IDs) who have four or more meaningful posts (ie, posts with five or more words) in the specific subreddit. In a different study [78], a similar threshold was used to determine lurkers who are not yet regularly contributing members. We used a similar threshold to identify regular members. We summarize the OHC dataset in Table 1.

The research reported in this study was exempted from review by the University of Utah’s institutional review board (IRB; ethics committee; IRB 00076188) under Exemption 2 as defined in US Federal Regulations 45 CFR 46.101(b).

### Research Question 1: Analysis for Communication Challenges in Social Media

To understand how language impairment manifests in written communication, we first measure the readability of posts. Readability of posts assesses writers’ ability to simply and clearly present ideas. To assess readability, we used *Flesch-Kincaid grade level* [60], *Simple Measure of Gobbledygook (SMOG) index* [59], *Gunning Fog index* [39], and *Linsear Write formula* [61], all of which are widely used metrics in readability studies [47-56]. Even though readability metrics have been shown to correlate with one another [46], different readability metrics can still generate a range of results. To increase the reliability of our results, we calculated the mean of the four readability metrics, following the procedures of previous studies [47,48]. Additionally, we used *min-max normalization* in our analyses to give equal weight to each readability metric (*readability score* from here on out to maintain clarity); however, we also report the complete readability results by each readability metric and the mean before the normalization. To automatically perform the readability analysis, we used the open-source Python *textstat* package [79].

To calculate the mean of readability scores for each subreddit, we first calculated the mean of readability scores for individual members, then we calculated the mean for each subreddit. Next, we normalized the mean of readability scores for individual members based on minimum and maximum values of the specific communities. This two-step process is to prevent one prolific member skewing the mean of a subreddit. We then measured the lexical diversity by calculating the percent of unique words in posts (ie, the number of unique words divided by the number of total words) with the same two-step process, excluding the normalization process.

To compare readability scores and lexical diversity among different subreddits, we first conducted pairwise independent sample *t* tests, followed by *P* value adjustments using the prespecified Hommel procedure [80] to adjust for multiple comparisons. Given the large sample of members, we also reported effect sizes (*d*) using Cohen *d* [81], as well as 95% CI for the pairwise comparisons, following suggestions of a previous study [82]. The effect sizes were interpreted as *d* (.01)=very small, *d* (.2)=small, *d* (.5)=medium, *d* (.8)=large, *d* (1.2)=very large, and *d* (2.0)=huge [81,83]. We used the open-source R *lsr* package to measure the effect size [84].

To bolster our findings, we manually examined the validity of using readability scores for the purpose of measuring communication challenges. Because high readability scores can also indicate sophisticated language with complex sentence structure, we manually analyzed a randomly selected sample of 120 posts (ie, 20 posts from each subreddit) after controlling for the post lengths and readability scores: 60 posts with high readability scores (ie, top 5% readability scores of a respective subreddit) and 60 posts with low readability scores (ie, bottom 5% readability scores of a respective subreddit). Furthermore, we manually assigned these posts into high and low readability groups to compare readability scores against manual judgments.

### Research Question 2: Analysis for Change of Communication Over Time in Social Media

To measure the change of readability and lexical diversity of posts made by each member participating in the six subreddits, we first calculated the readability scores and lexical diversity of individual posts. Then, we organized each post’s readability score and lexical diversity according to the posting time per-member basis for the subreddit. Next, we applied linear least squares regression to them against the interaction sequence (ie, determined by the posting time) for each member. We performed linear least squares regression against the interaction sequence rather than time because we are interested in the change caused by each interaction rather than time. We reported the mean of slopes for readability scores and lexical diversity to reflect the overall changes in members in each of the six subreddits. Next, we applied pairwise independent sample *t* tests and the Hommel procedure. We then reported effect sizes and 95% CIs as we did in RQ1. For both analyses, we also reported a comparison among r/happy, r/loseit, and r/bodybuilding to deepen our understanding of the effects of emotional challenges in language impairment.

**Table 1:** Summary of the dataset.

Subreddit	Dates	Number of posts	Number of members
r/depression	December 2008 to May 2015	526,470	34,685
r/bipolar	January 2010 to May 2015	146,328	5019
r/schizophrenia	October 2012 to May 2015	22,273	896
r/happy	January 2008 to May 2015	70,516	6433
r/loseit	July 2010 to May 2015	1,054,949	46,367
r/bodybuilding	August 2009 to May 2015	724,190	18,927

## Results

### Research Question 1: Analysis for Communication Challenges in Social Media

**Figure 1:**
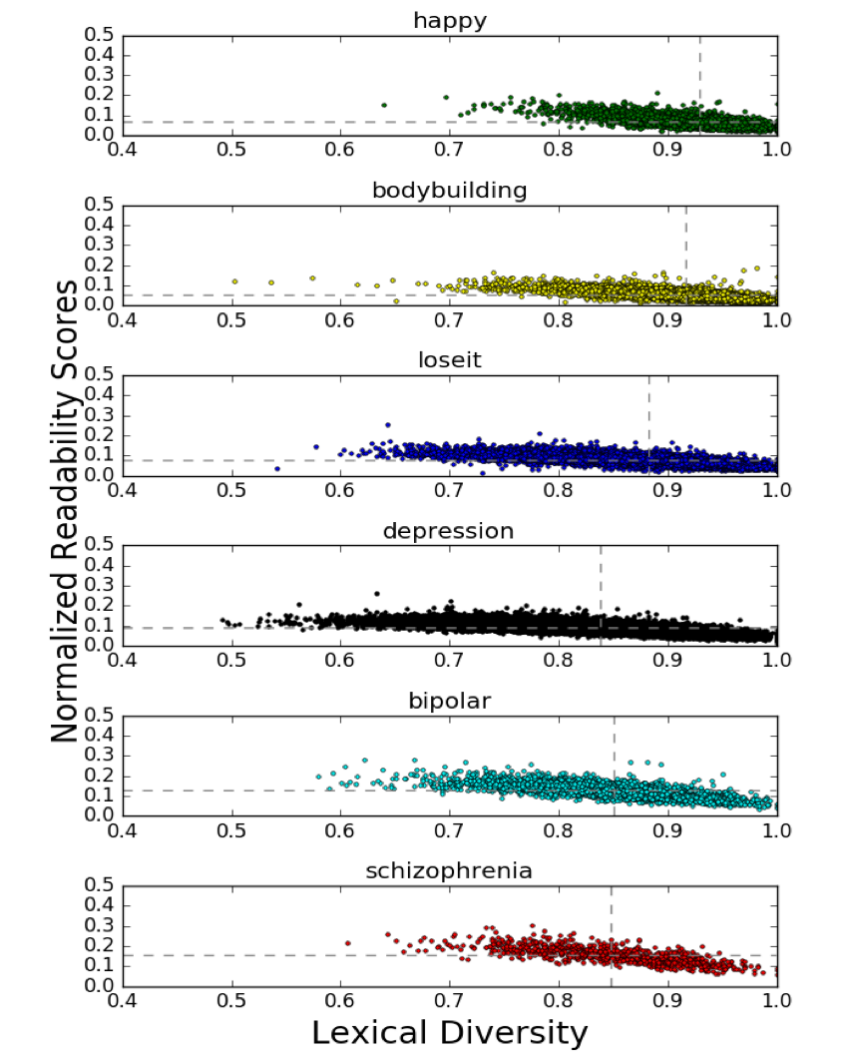
An overview of mean readability scores and lexical diversity among the six subreddits. The gray dotted lines represent the mean of the axes.

We captured the mean and SE for (1) individual readability scores measured by four different metrics, (2) mean readability scores of the four metrics, (3) normalized mean readability scores of the four metrics, (4) lexical diversity, and (5) the total number of words in posts for each of the five communities (Table 2). On average, posts from r/schizophrenia were found to be the most difficult to read (ie, highest normalized readability scores), followed by posts from r/bipolar, r/depression, r/loseit, r/happy, and then r/bodybuilding. Lexical diversity showed a similar trend. On average, posts from r/happy had the most diverse lexicon, followed by posts from r/bodybuilding, r/loseit, r/bipolar, r/schizophrenia, and then r/depression. Figure 1 presents a scatter plot of the mean readability scores and lexical diversity among six different subreddits.

We then conducted pairwise independent sample *t* tests to compare readability scores and lexical diversity of each subreddit to understand the differences between two subreddits. Pairwise comparisons of normalized readability scores among subreddits are shown in Table 3.

Posts from r/bodybuilding, r/happy, and r/loseit were statistically significantly more simply written than posts from r/depression, r/bipolar, and r/schizophrenia in terms of syntactic complexity and word frequency that were measured in readability. The effect sizes were also in between medium to huge when readability scores of r/happy, r/loseit, and r/bodybuilding were compared to readability scores of r/depression, r/bipolar, and r/schizophrenia. Table 3 summarizes these findings.

Pairwise comparisons of lexical diversity showed similar results (Table 4). Posts from r/happy and r/bodybuilding used a significantly more diverse lexicon than the posts from r/depression, r/bipolar, and r/schizophrenia. The effect sizes ranged between very large to huge. Posts from r/loseit also had a significantly more diverse lexicon and had medium to large effect sizes than the posts from the three mental health subreddits. Differences in lexical diversity among posts from the three mental health subreddits had very small to small effect sizes. The lexical diversity differences between posts from r/bipolar and r/depression, as well as between r/schizophrenia and r/depression were statistically significant; however, posts from r/bipolar and r/schizophrenia were not significantly different. Interestingly, a significant difference with large to very large effect size of lexical diversity was found between the posts from r/happy and r/loseit as well. Table 4 summarizes findings on lexical diversity differences.

In our manual analyses, we found that both high and low readability score posts resembled common internet communication and was void of sophisticated writing. However, we encountered several inadequately articulated posts, many in the form of run-on sentence structure. Using inadequate articulation as a guide, we manually assigned 120 posts into high or low readability groups. The manual assessment agreed with the readability score 68% of the time (82 out of 120). The readability score and manual assessment had higher agreement in posts from mental health subreddits compared with the control groups. Mental health subreddits, r/depression, r/bipolar, and r/schizophrenia, had 80%, 80%, and 90% agreement, respectively. Conversely, the control subreddits, r/happy, r/loseit, and r/bodybuilding, had 40%, 70%, and 50% agreement, respectively.

### Research Question 2: Analysis for Change of Communication Over Time in Social Media

To understand the effects of participating in online mental health communities with respect to their written communication, we applied linear least squares regression to readability scores and lexical diversity against the interaction sequence.

Members of the three mental health subreddits showed improvement in both readability scores (ie, negative slope for improvement) and lexical diversity (ie, positive slope for improvement). Among the mental health subreddits, r/bipolar showed the most improvement, followed by r/depression and r/schizophrenia for readability scores. For lexical diversity, members improved in order of r/bipolar, r/schizophrenia, and then r/depression. Members of r/bodybuilding had the biggest improvement in readability scores, and members of r/loseit also improved in both readability scores and lexical diversity. Members of r/happy only improved in lexical diversity (Table 5).

**Table 2:** Communication challenges in members. Variables are reported as the mean (SE) of readability scores, normalized mean of readability scores, lexical diversities, and the total number of words in posts for each community. SMOG: Simple Measure of Gobbledygook.

Subreddit	Flesch-Kincaid grade, mean (SE)	SMOG index, mean (SE)	Gunning Fog index, mean (SE)	Linsear Write formula, mean (SE)	Four metrics, mean (SE)	Four metrics, normalized mean (SE)	Lexical diversity, mean (SE)	Total number of words in posts, mean (SE)
r/happy	4.83 (0.03)	1.61 (0.02)	16.76 (0.03)	5.22 (0.02)	7.11 (0.02)	0.06 (0.0003)	0.93 (0.001)	29.10 (0.25)
r/bodybuilding	5.03 (0.02)	1.92 (0.01)	17.21 (0.02)	5.90 (0.01)	7.51 (0.01)	0.05 (0.0001)	0.92 (0.0003)	34.01 (0.16)
r/loseit	4.83 (0.01)	2.90 (0.01)	16.76 (0.01)	6.06 (0.01)	7.64 (0.01)	0.08 (8.3e-05)	0.88 (0.0003)	52.37 (0.16)
r/depression	5.53 (0.01)	3.74 (0.01)	17.05 (0.01)	6.69 (0.01)	8.25 (0.01)	0.09 (0.0001)	0.84 (0.0004)	76.24 (0.29)
r/bipolar	5.88 (0.03)	3.92 (0.02)	17.80 (0.03)	6.72 (0.03)	8.58 (0.02)	0.13 (0.0004)	0.85 (0.001)	69.76 (0.62)
r/schizophrenia	6.67 (0.08)	4.17 (0.07)	18.55 (0.08)	7.26 (0.09)	9.16 (0.06)	0.16 (0.001)	0.85 (0.002)	72.10 (1.65)

**Table 3:** Pairwise *t* test of the normalized average scores of four metrics.

Subreddit Comparison (ordered by readability scores)		*t* value	*P* value	Adjusted *P* value (Hommel)	95% CI	Effect size (*d*)
**r/schizophrenia**							
	vs r/bipolar	22.52	<.001	<.001	0.03-0.03	0.97 (large-very large)
	vs r/depression	52.70	<.001	<.001	0.07-0.07	3.18 (huge)
	vs r/loseit	62.71	<.001	<.001	0.08-0.08	4.43 (huge)
	vs r/happy	68.90	<.001	<.001	0.09-0.09	3.46 (huge)
	vs r/bodybuilding	81.41	<.001	<.001	0.10-0.11	5.64 (huge)
**r/bipolar**							
	vs r/depression	85.94	<.001	<.001	0.04-0.04	1.70 (very large-huge)
	vs r/loseit	116.99	<.001	<.001	0.05-0.05	2.62 (huge)
	vs r/happy	116.71	<.001	<.001	0.06-0.06	2.26 (huge)
	vs r/bodybuilding	169.66	<.001	<.001	0.07-0.08	3.64 (huge)
**r/depression**							
	vs r/loseit	92.43	<.001	<.001	0.01-0.01	0.67 (medium-large)
	vs r/happy	71.60	<.001	<.001	0.02-0.02	1.08 (large-very large)
	vs r/bodybuilding	222.10	<.001	<.001	0.04-0.04	1.90 (very large-huge)
**r/loseit**							
	vs r/happy	32.69	<.001	<.001	0.01-0.01	0.55 (medium-large)
	vs r/bodybuilding	163.41	<.001	<.001	0.02-0.02	1.39 (very large-huge)
**r/happy**							
	vs r/bodybuilding	43.57	<.001	<.001	0.01-0.01	0.74 (medium-large)

**Table 4:** Pairwise *t* test of lexical diversity.

Subreddit Comparison (ordered by readability scores)		*t* value	*P* value	Adjusted *P* value (Hommel)	95% CI	Effect size (*d*)
**r/happy**							
	vs r/bodybuilding	20.83	<.001	<.001	0.01 to 0.01	0.30 (small-medium)
	vs r/loseit	80.58	<.001	<.001	0.05 to 0.05	0.89 (large-very large)
	vs r/bipolar	80.04	<.001	<.001	0.08 to 0.08	1.57 (very large-huge)
	vs r/schizophrenia	36.89	<.001	<.001	0.08 to 0.09	1.80 (very large-huge)
	vs r/depression	143.16	<.001	<.001	0.09 to 0.09	1.42 (very large-huge)
**r/bodybuilding**							
	vs r/loseit	84.41	<.001	<.001	0.03 to 0.03	0.66 (medium-large)
	vs r/bipolar	74.10	<.001	<.001	0.06 to 0.07	1.40 (very large-huge)
	vs r/schizophrenia	31.70	<.001	<.001	0.06 to 0.07	1.54 (very large-huge)
	vs r/depression	163.08	<.001	<.001	0.08 to 0.08	1.30 (very large-huge)
**r/loseit**							
	vs r/bipolar	36.78	<.001	<.001	0.03 to 0.03	0.59 (medium - large)
	vs r/schizophrenia	16.09	<.001	<.001	0.03 to 0.04	0.64 (medium-large)
	vs r/depression	100.61	<.001	<.001	0.04 to 0.05	0.74 (medium-large)
**r/bipolar**							
	vs r/schizophrenia	1.21	.23	.23	−0.002 to 0.01	0.05 (very small-small)
	vs r/depression	13.63	<.001	<.001	0.01 to 0.01	0.19 (very small-small)
**r/schizophrenia**							
	vs r/depression	4.41	<.001	<.001	0.01 to 0.01	0.14 (very small-small)

**Table 5:** Writing quality changes in members. Variables are reported as the mean (SE) of slopes for readability scores, normalized mean of slopes for readability scores, slope of lexical diversities, and slope of the total number of words in posts for each community. SMOG: Simple Measure of Gobbledygook.

Subreddit	Flesch-Kincaid grade, mean (SE)	SMOG index, mean (SE)	Gunning Fog index, mean (SE)	Linsear Write formula, mean (SE)	Four metrics, mean (SE)	Four metrics, normalized mean (SE)	Lexical diversity, mean (SE)	Total number of words in posts, mean (SE)
r/happy	−0.01 (0.005)	0.0002 (0.01)	−0.004 (0.003)	−0.01 (0.01)	−0.01 (0.01)	0.13 (0.56)	0.34 (0.23)	−0.002 (0.001)
r/bodybuilding	−0.05 (0.01)	−0.21 (0.10)	−0.06 (0.04)	−0.29 (0.22)	−0.10 (0.03)	−15.22 (5.17)	3.85 (0.34)	−0.01 (0.001)
r/loseit	−0.002 (0.005)	−0.08 (0.04)	−0.02 (0.02)	−0.02 (0.01)	−0.03 (0.01)	−5.37 (1.74)	0.93 (0.88)	−0.005 (0.0006)
r/depression	−0.03 (0.004)	−0.06 (0.004)	−0.01 (0.003)	−0.04 (0.004)	−0.05 (0.005)	−5.59 (0.32)	2.40 (0.10)	−0.01 (0.0003)
r/bipolar	−0.09 (0.01)	−0.14 (0.02)	−0.03 (0.01)	−0.12 (0.02)	−0.14 (0.02)	−9.12 (1.02)	5.04 (0.48)	−0.01(0.001)
r/schizophrenia	−0.02 (0.02)	−0.09 (0.02)	0.005 (0.02)	−0.07 (0.04)	−0.07 (0.03)	−4.69 (1.25)	3.89 (0.89)	−0.01 (0.002)

**Table 6:** Pairwise *t* test of changes of the normalized average readability scores.

Subreddit Comparison (ordered by readability scores)		*t* value	*P* value	Adjusted *P* value (Hommel)	95% CI	Effect size (*d*)
**r/happy**						
	vs r/schizophrenia	3.52	<.001	.01	2.13 to 7.51	0.11 (very small-small)
	vs r/loseit	3.01	.003	.03	1.92 to 9.08	0.02 (very small-small)
	vs r/depression	8.87	<.001	<.001	4.46 to 6.99	0.10 (very small-small)
	vs r/bipolar	7.98	<.001	<.001	6.98 to 11.53	0.16 (very small-small)
	vs r/bodybuilding	2.95	.003	.03	5.17 to 25.54	0.02 (very small-small)
**r/schizophrenia**						
	vs r/loseit	0.32	.75	.90	−3.52 to 4.88	0.002 (very small)
	vs r/depression	0.70	.49	.90	−1.64 to 3.44	0.02 (very small-small)
	vs r/bipolar	2.75	.01	.05	1.27 to 7.59	0.07 (very small-small)
	vs r/bodybuilding	1.98	.05	.24	0.11 to 20.95	0.02 (very small-small)
**r/loseit**						
	vs r/depression	0.12	.90	.90	−3.25 to 3.69	0.0008 (very small)
	vs r/bipolar	1.86	.06	.31	−0.20 to 7.70	0.01 (very small-small)
	vs r/bodybuilding	1.81	.07	.35	−0.84 to 20.53	0.02 (very small-small)
**r/depression**						
	vs r/bipolar	3.31	.001	.01	1.44 to 5.62	0.06 (very small-small)
	vs r/bodybuilding	1.86	.06	.31	−0.52 to 19.77	0.02 (very small-small)
**r/bipolar**						
	vs r/bodybuilding	1.16	.25	.90	−4.22 to 16.42	0.01 (very small)

To understand the significance of the changes in readability scores and lexical diversity, we compared the changes that occurred in the three mental health subreddits against r/happy, r/bodybuilding, and r/loseit via pairwise independent sample *t* tests. The overall comparisons of readability scores among subreddits are shown in Table 6.

Subreddit comparisons indicate that the readability of posts by members of all three mental health subreddits improved significantly more than members of r/happy. Yet, the effect sizes for those comparisons were very small to small. Moreover, only the readability of posts by members of r/bipolar improved significantly more than posts by members of r/depression and r/schizophrenia, with very small to small effects among the pairwise comparison of three mental health subreddits.

Members of r/bipolar also had the most improvement in terms of lexical diversity and significantly more than members of r/depression, r/loseit, and r/happy, albeit the effect sizes were very small to small (Table 7). Furthermore, members of r/schizophrenia and r/depression improved significantly more than members of r/happy; however, no significant difference was found against r/loseit.

**Table 7:** Pairwise *t* test of lexical diversity changes.

Subreddit Comparison (ordered by readability scores)		*t* value	*P* value	Adjusted *P* value (Hommel)	95% CI	Effect size (*d*)
**r/bipolar**						
	vs r/schizophrenia	1.14	.25	.76	−0.83 to 3.13	0.03 (very small-small)
	vs r/bodybuilding	2.01	.04	.22	0.03 to 2.36	0.03 (very small-small)
	vs r/depression	5.35	<.001	<.001	1.68 to 3.61	0.12 (very small-small)
	vs r/loseit	4.09	<.001	<.001	2.14-6.08	0.02 (very small-small)
	vs r/happy	8.77	<.001	<.001	3.65 to 5.75	0.18 (very small-small)
**r/schizophrenia**						
	vs r/bodybuilding	0.04	.96	.96	−1.82 to 1.91	0.001 (very small)
	vs r/depression	1.68	.09	.38	−0.26 to 3.24	0.08 (very small-small)
	vs r/loseit	2.37	.02	.12	0.51 to 5.41	0.02 (very small-small)
	vs r/happy	3.88	<.001	<.001	1.75 to 5.35	0.18 (very small-small)
**r/bodybuilding**						
	vs r/depression	4.05	<.001	<.001	0.75 to 2.15	0.05 (very small-small)
	vs r/loseit	3.08	.002	.02	1.06 to 4.77	0.02 (very small-small)
	vs r/happy	8.45	<.001	<.001	2.69 to 4.32	0.08 (very small-small)
**r/depression**						
	vs r/loseit	1.66	.10	.39	−0.27 to 3.21	0.01 (very small-small)
	vs r/happy	8.16	<.001	<.001	1.56 to 2.55	0.11 (very small-small)
**r/loseit**						
	vs r/happy	0.64	.52	.96	−1.2 to 2.37	0.003 (very small)

## Discussion

### Principal Findings

We examined the issue of written communication challenges using readability and lexical diversity of posts from publicly accessible online mental health communities on Reddit. We found that on average, members of depression, bipolar disorder, and schizophrenia subreddits wrote posts that are significantly more difficult to read and had significantly less lexical diversity when compared with three other OHCs focusing on positive emotion, exercising, and weight management.

We also found that as members of mental health communities participated more in the community, they wrote posts that were easier to read with more lexical diversity. Interestingly, members of other OHCs also improved, with the exception of readability scores of r/happy members. Only r/bipolar members showed statistically significant improvement in lexical diversity compared with members of the two other OHCs (r/happy and r/loseit), while showing statistically significant improvement compared with r/happy in terms of readability scores. Compared with r/happy members, r/depression and r/schizophrenia members also significantly improved in both examined features.

Another interesting finding is readability scores and lexical diversity of r/loseit, in which members could have depressive symptoms because of the distress of being overweight. The readability scores and lexical diversity of r/loseit were in between r/happy and three mental health subreddits. Still, the posts from r/loseit were statistically significantly easier to read with more lexical diversity (medium to huge effect sizes) compared with the three mental health subreddits. However, posts from r/loseit were statistically significantly harder to read (medium to large effect size), with less lexical diversity (large to very large effect size) compared with r/happy. Members of r/bodybuilding and r/happy wrote more similar to one another than to members of r/loseit in terms of readability scores and lexical diversity.

Despite the possible language impairment faced by members of mental health communities, their real-life communication challenges are unknown. To our knowledge, this is the first study to show mental health community members’ written communication challenges occurring in the real world using social media.

### Practical Implication for Online Communication and Mental Health

Our analyses suggest that members of online mental health communities could encounter incoherent texts because of the language impairment of other members. Automatically correcting misspellings [85], simplifying language [86], and improving text coherence [87] in posts could enhance the readability of posts and the overall experience of participating in these communities.

Many online communities, including many Reddit’s subreddits, utilize moderators to regulate content and support members. A number of automated systems have been suggested to assist moderators and reduce moderator burden [88]. Similarly, an adaptation of our automatic analysis method could be a basis for detecting individuals whose lexical diversity and readability of posts are worsening in massive scale networks. This could indicate worsening of mental disorder symptoms, and such a feature could alert and allow moderators to provide timely support.

We also showed the potential for improving written communication via more frequent writing in online mental health communities. Designing features of online mental health communities for the purpose of improving written communication can enhance the everyday life of individuals suffering from mental conditions. For example, a place for expressive writing can improve their symptoms [89,90] and possibly help with their written communication challenges.

### User Privacy

Research using publicly accessible social media data (such as Reddit) is typically granted exemption from review by IRBs in the US context; however, ethical considerations such as privacy remain critical [91-93]. In this paper, we do not report any user identifiable information to protect user privacy (eg, direct quotations and usernames).

### Limitation and Future Directions

Our study has several limitations. A number of confounding factors such as individuals’ premorbid-intelligence, -verbal skill and -education level, as well as demographic and geographical characteristics [9] could influence the writing quality other than language impairment. Other possible confounding factors associated with group dynamics and mental health conditions include the communication practices and cultures of specific subreddits, as well as medication and substance use of individuals suffering from mental health conditions. Furthermore, we assumed that high readability scores are reflecting inadequate articulation or organization by writers. Although inadequate articulation and organization can increase readability scores, high readability scores can also be because of sophisticated language and complex sentence structure. However, we did not encounter sophisticated writing in our manual assessment, and it is unlikely that such sophistication and complexity are highly prevalent in everyday communication. Similarly, we do not know how readability scores were influenced by common online communication attributes such as slang, abbreviation, community nomenclature, and misspellings [85], or how lexical diversity was impacted by number of topics and change of topics [94]. However, these online communication attributes are more likely to occur in all subreddits, and thus, affecting the readability scores in a similar manner. Reddit is a widely used platform more frequently used by young males [95,96] in English-speaking nations [96]. Despite more user activities from English speaking nations (85%) [96], it is unclear how participation by English as second language speakers is affecting the results. Additionally, members who choose to participate in r/depression, r/bipolar, and r/schizophrenia are not necessarily representative of their respective populations and are subject to selection bias. Similarly, we do not have any evidence that members of these three mental health subreddits are clinically diagnosed; the severity of their condition is unknown, and overlapping memberships could exist in these subreddits. However, one of the main limitations of previous studies were small sample sizes [7,8], which could be the underlying reason for the inconsistent results [10,23,24]. Thus, given the size of r/depression, r/bipolar, and r/schizophrenia, the prevalence and gravity of mental disorders, the increasing popularity of social media, and the potential challenges associated with daily use of social media make Reddit an interesting platform to study.

Although beyond the scope of this study, further investigation regarding readability metrics may be needed for more accurately determining the grade reading level [46]. We selected readability metrics based on the literature in which the metrics have been validated or used [44,46-56]. However, we noticed a disparity among the metrics. For example, readability scores by *Gunning Fog index* were far greater than the other three metrics. *SMOG index* resulted in readability scores that were less than the other metrics. Despite the apparent differences, the scores were correlated with one another as a previous study suggested [46], and we used the mean of normalized scores of four readability metrics to strengthen the reliability of our findings. Due to the consistent statistical results, we believe that these four metrics can measure the general difficulty of readability. We also acknowledge that our large sample size could have inflated the statistical significance levels. Thus, we reported 95% CIs and used effect sizes when interpreting the results. Another interesting future direction would be to investigate why members are improving and longitudinal changes in written communication with respect to prolonged participation in online mental health communities. In this study, we only examined the overall impact of participation in online mental health communities; however, understanding how members are improving their written communication skills could potentially inform the design of related patient education programs.

### Conclusions

We provide new insights into the written communication challenges faced by individuals suffering from depression, bipolar disorder, and schizophrenia. A comparison of mental health communities to three other OHCs suggests that writings in mental health communities were significantly more difficult to read, while consisting of a significantly less diverse lexicon. Our findings also suggest that participating in these subreddits has the potential to improve members’ written communication over time. We contribute practical suggestions for utilizing our findings in online communication settings to enhance members’ communicative experience. We consider these findings to be an important step toward understanding written communication challenges among individuals suffering from mental disorders.

## Abbreviations

IRB: institutional review board
OHC: online health community
RQ: research question
SMOG: Simple Measure of Gobbledygook
